# Hyperspectral estimation of chlorophyll content in grapevine based on feature selection and GA-BP

**DOI:** 10.1038/s41598-024-84977-x

**Published:** 2025-03-07

**Authors:** YaFeng Li, XinGang Xu, WenBiao Wu, Yaohui Zhu, LuTao Gao, XiangTai Jiang, Yang Meng, GuiJun Yang, HanYu Xue

**Affiliations:** 1 Key Laboratory of Quantitative Remote Sensing in Ministry of Agriculture and Rural Affairs, Information Technology Research Center, Beijing Academy of Agricultural and Forestry Sciences, Beijing, 100097 China; 2 School of Agricultural Engineering, Jiangsu University, Zhenjiang, 212013 China; 3 College of Big Data, Yunnan Agricultural University, Yunnan, 650500 China

**Keywords:** Data preprocessing, Feature selection, Machine learning, Hyperspectral monitoring., Data processing, Machine learning

## Abstract

Leaf chlorophyll content (LCC) is a key indicator for assessing the growth of grapes. Hyperspectral techniques have been applied to LCC research. However, quantitative prediction of grape LCC using this technique remains challenging due to baseline drift, spectral peak overlap, and ambiguity in the sensitive spectral range. To address these issues, two typical crop leaf hyperspectral data were collected to reveal the spectral response characteristics of grape LCC using standardization by variables (SNV) and multiple far scattering correction (MSC) preprocessing variations. The sensitive spectral range is determined by Pearson’s algorithm, and sensitive features are further extracted within that range using Extreme Gradient Boosting (XGBoost), Recursive Feature Elimination (RFE), and Principal components analysis (PCA). Comparison of the prediction ability of Random Forest Regression (RFR) algorithm, Support Vector Machine Regression (SVR) model, and Genetic Algorithm-Based Neural Network (GA-BP) on grape LCC based on sensitive features. A SNV-RFE-GA-BP framework for predicting hyperspectral LCC in grapes is proposed, where $\:{R}^{2}$=0.835 and NRMSE = 0.091. The analysis results show that SNV and MSC treatments improve the correlation between spectral reflectance and LCC, and different feature screening methods have a greater impact on the model prediction accuracy. It was shown that SNV-based processed hyperspectral data combined with GA-BP has great potential for efficient chlorophyll monitoring in grapevine. This method provides a new framework theory for constructing a hyperspectral analytical model of grapevine key growth indicators.

## Introduction

Yunnan Province is located in the low-latitude plateau area, mainly by the South Bengal high-pressure air flow influence of the formation of the plateau monsoon climate. Most of the province has warm winters and cool summers, with four seasons of spring^1^. Yunnan grapes are mainly distributed at an altitude of 400–2800 m between these areas, the annual average temperature between 10 and 23.6$\:\:℃$, rainfall of 550–1200 mm, the number of hours of sunshine in 2000 h or more, the annual sunshine rate is greater than 45%, so most of the province’s regions are suitable for planting grapes^2,3^. Photosynthesis is the most basic and important function of grapevine leaves^4^. The main site of photosynthesis in green plants is the chloroplast, which contains chlorophyll, the main photosynthetic pigment. Therefore, leaf chlorophyll content (LCC) plays a crucial role in grape growth and yield which is an important indicator for fruit growers to manage their vineyards^5,6^. It can directly reflect the growth condition of the plant, when LCC is too low, the senescence of leaves will affect the synthesis of organic nutrients, resulting in grapes without coloring or grapes that have been fully colored to appear soft fruit and drop grains, which directly affects the quality of the fruit^7^. It can also be used as an approximate estimate of leaf nitrogen concentration, which provides an important indicator for crop growth evaluation, yield estimation, and monitoring of pests and diseases^8^.

The traditional laboratory chemical methods for analyzing LCC methods are methodologically complex, resulting in time-consuming and can cause damage to the leaves^9^. A handheld portable chlorophyll meter can determine the relative chlorophyll content, SPAD- 502 plus is a handheld soil and crop analyzer developed in Japan and is widely used worldwide^10^. However, for precision agriculture, a single content can only be obtained through point-by-point measurements, and real-time monitoring of plant variables cannot be realized. In recent years, hyperspectral remote sensing technology has been developing rapidly, and hyperspectral equipment provides a fast, nondestructive, and timely method of data collection, which can be used to measure the nutrient status of crops and to determine the growth status of plants. The use of spectroscopic techniques to detect crop chlorophyll^11^, biomass^12^ and yield^13^ has become a hot topic. Wang et al.^14^ predicted the chlorophyll content of winter wheat at each fertility stage based on full-band in situ hyperspectral data, combined with Ridge regression (Ridge), gradient regression counting algorithm (GRBT) and other models. Lin et al.^15^ Modelled N, P, and K status of summer maize by in situ canopy hyperspectral data. An et al.^16^ estimated wheat canopy powdery mildew based on in situ hyperspectral response and feature screening. Most of the above studies used in situ hyperspectral data to predict biochemical indicators of crop growth and achieved better results.

However, leaf chlorophyll spectral response curves are affected by a number of factors, including leaf water content, carotenoid and flavonoid compounds, and baseline drift caused by the measuring instrument^17^. Chlorophyll has sensitive bands in the visible and near-infrared wavelength ranges, but the presence of other compounds interferes with the light signals in these wavelength ranges, and when the absorption bands overlap with chlorophyll, it can be difficult to extract the sensitive bands of LCC^18,19^. Few in-depth studies have been reported on the challenges associated with LCC estimation in grapes, and how to minimize the effects of spectral overlap and baseline drift, extracting chlorophyll-sensitive segments is a necessary prerequisite for improving the accuracy of chlorophyll estimation. Standard normal variate (SNV) and multiplicative scatter correction (MSC) are commonly used algorithms for the preprocessing of hyperspectral data, which can effectively eliminate the spectral differences due to different scattering levels^20^. Cui et al.^21^ monitored early ulcers in apples based on hyperspectral imaging and found that MSC with processed spectra combined with a dual-channel convolutional neural network (DC-CNN) model performed the best, with 98% accuracy. Lu et al.^22^ used chlorophyll fluorescence (ChlF) induction curves and hyperspectral images to assess leaf nitrogen content (LNC), and the results showed that the model using SNV produced the best performance. All the above studies have shown that SNV and MSC can correct the baseline drift phenomenon of spectral data by ideal spectra, thus enhancing the correlation between spectra and data.

High-dimensional hyperspectral data contain redundant information unrelated to the response variable, and the contradiction between the computational intensity and accuracy of sensitive band selection is also a problem that cannot be ignored for feature screening methods^23,24^. The Pearson correlation coefficient is a standardized statistic that is constructed based on covariance and standard deviation which can help to screen out independent variables with significant linear relationships^25^. Sahoo et al. extracted LCC strong correlation bands based on Pearson’s algorithm to correlate UAV spectra with farmland traits^26^. However, there are still high correlations between some sensitive bands, and it is crucial to improve the computational efficiency of the model and simplify the model structure while maximizing the retention of spectral information^27^. Extreme gradient boosting (XGBoost) is a tree-based algorithm for efficient and fast implementation of the gradient boosting decision tree (GBDT) algorithm. Decision trees are created iteratively by splitting the features and generating a new function for each tree to model the residuals of the previous predictions, and the importance scores of the feature variables are computed for feature selection while training the model^28^. Zou et al. demonstrated the effectiveness of XGBoost feature extraction by encoding and reconstructing the XGBoost leaf node feature information to obtain implicit features of the original data in the NIR spectra, and combining it with convolutional neural network (CNN) for the prediction of maize multicomponent^29^. Recursive feature elimination (RFE) can effectively filter out the set of features that are most valuable for the prediction of the target variable by setting a specified number of features by iteratively constructing the model and eliminating the least important features each time^30^. Fu et al. proposed a mangrove species mapping method based on the combination of RFE feature selection algorithm combined with deep learning (DL), and evaluated the classification ability of the RFE-DL Suna method by taking advantage of the UAV multispectral images, which proved the feature selection ability of RFE in the multidimensional dataset^31^. Principal component analysis (PCA) can project the original data onto selected principal components to reduce the data dimensionality and improve the efficiency and generalization of the algorithm without losing too much information^32^. Ji et al. converted the raw data into several new, relatively independent, and comprehensive indices by PCA and analyzed them in combination with the affiliation function, and found that the method could evaluate plant stress tolerance more comprehensively and objectively^33^.

The core of regression predictive modeling is to learn the input-to-output mapping relationship, mapping the feature matrix of a sample to the sample label space, so a reasonable model selection is also a key factor affecting the modeling accuracy^34,35^. Random forests (RFR) and neural networks (BP), as classical machine learning (ML) models, have been widely used in precision agriculture^36,37^. Yang et al.^38^ compared the ability of several machine learning methods to predict chlorophyll-a in rivers with different hydrological characteristics, and the results showed that the RF model outperformed the support vector machine regression (SVR) model. Qi et al.^39^ conducted feature extraction based on UAV multispectral imagery to monitor peanut LCC and found that BP network is the most suitable model to monitor peanut LCC with better fit and accuracy than RF. Genetic algorithm (GA) replaces the back-propagation process in BP by the operations of selection, crossover, and mutation, which allows for higher predictive power^40,41^. All of the above studies demonstrated the excellent performance of RFR, GA-BP, and SVR models in regression prediction, so the above three models were selected for use as grape LCC prediction in this study.

The aim of this study is to develop a model for LCC estimation from remote sensing spectra of grapes with generalization. By applying SNV and MSC preprocessing changes to the in situ hyperspectral data, effects such as baseline drift caused by the remaining compounds and instrumentation on the spectral curves are reduced. The LCC response band range is initially determined by Pearson correlation analysis, in which the sensitive features are further screened by XGBoost, RFE, and PCA to reduce the input dimension and improve the model efficiency. Based on three typical machine learning algorithms, GA-BP, RFR, and SVR, the capability of different algorithms in the spectral monitoring of grape LCC is investigated to provide methodological references for the nondestructive monitoring and diagnosis of grape leaf nutrient spectra.

## Materials and methods

### Experimental area

**Fig. 1:**
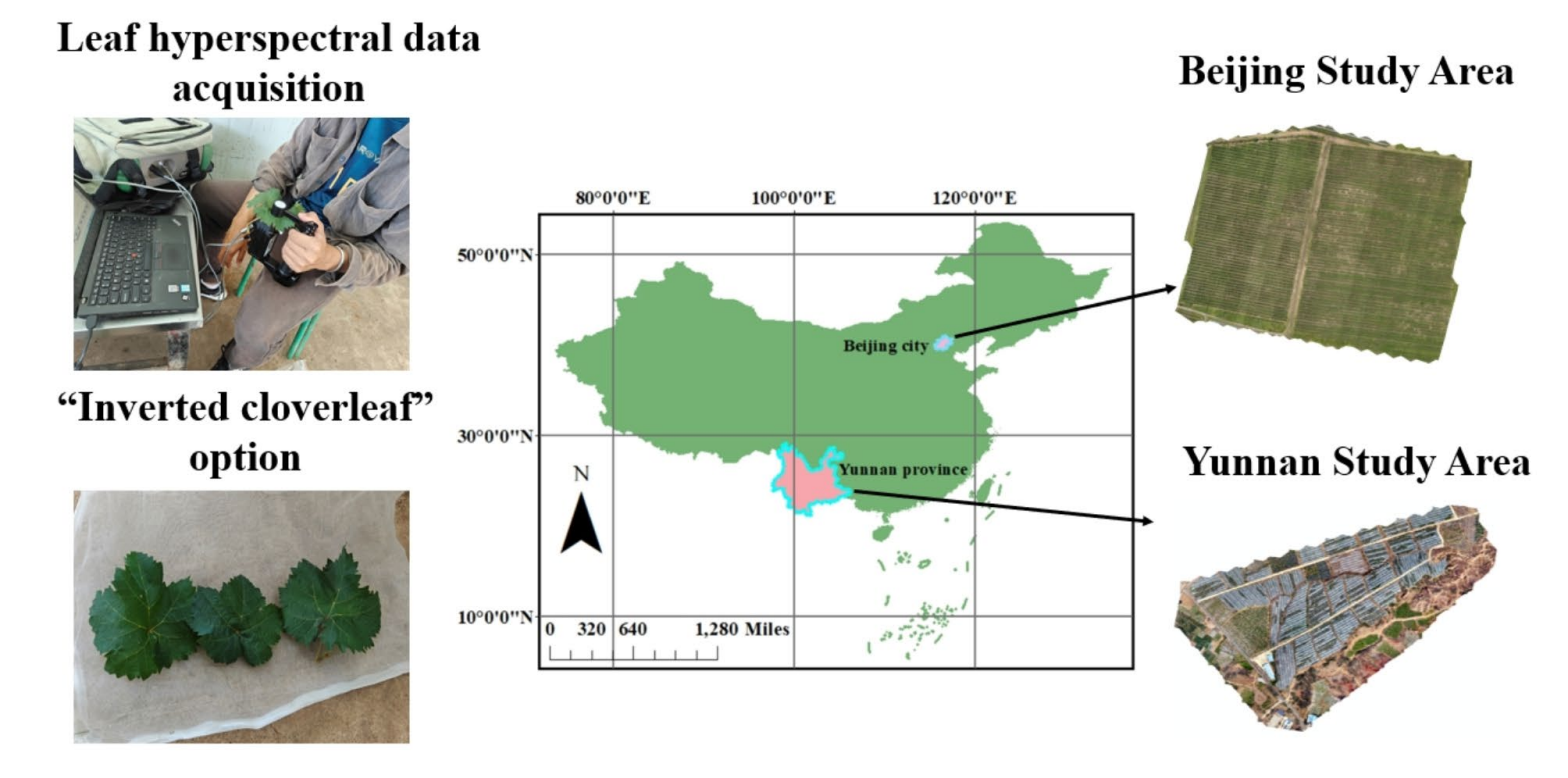
Study area.

Figure 1 shows the experimental area of this study. The Cabernet Sauvignon experimental area is located in Bolongbao Vineyard, Fangshan District, Beijing, which is situated between the longitude of 131.45-132.2$\:^\circ\:$E and latitude of 46.47–47.0$\:^\circ\:$N. The winegrowing area is located in the “Golden Line of Wine Grape Growing” at 40 degrees north latitude, accompanied by the Wulan Mountain in the west and the ancient channel of the Dashi River in the east, which has a microclimate of “mountain front warm zone”. The old Boulder River Road provides excellent gravel soils, while the richness of the volcanic rocks and the favorable slope alignment make this an excellent place to grow grapes. The data were collected on 2022.08.19, the phenological period was maturity, and there were 32 sample points.

The Edible Grapes Sunshine Rose Experimental Area is located at Dongfanghong Farm, Yuanmou County, Chuxiong Yi Autonomous Prefecture, Yunnan Province, which is situated at a longitude of 25.75$\:^\circ\:$E and a latitude of 101.77$\:^\circ\:$N. It belongs to the northern tropical to southern subtropical hot and dry monsoon climate, in a year, wet and dry, hot and dry climate, long summer and no winter, small difference in temperature yearly, large difference in temperature daily, sunny days, light quality is good, belongs to the high sunshine area, for the sunshine rose grapes to create a good environment for growth. The collection dates were 2023.08.23 and 2023.11.04, and the phenological periods were the berry growth and ripening periods, with 30 and 29 sample sites.

Plants are able to detect subtle changes in the quality, light, duration and direction of light intensity in the environment in which they grow, thus causing changes in the physiological and morphological structures necessary for survival in that environment. There are obvious differences in crop varieties and growing environments between the two regions, so this study used the above region as the study area, aiming to establish a generalized model for grape LNC prediction.

### Data acquisition

#### Leaf chlorophyll determination

Traditional methods for chlorophyll determination generally use spectrophotometry, which is time-consuming and damages the crop at the same time. However, studies have shown that soil and plant analyzer development (SPAD) and chlorophyll content has a significant correlation, SPAD value can better reflect the changes in leaf chlorophyll content^42^. The use of chlorophyll meter to determine the chlorophyll content of leaves is completely feasible, under certain conditions can be used instead of the direct determination of chlorophyll content. In this study, three grape “inverted trifoliate leaves” were selected from each plot, which are considered to be the most functional leaves, and their growth condition plays a decisive role in the yield and quality of grapes^43^. Chlorophyll SPAD values were determined using a SPAD-502plus (Konica Minolta) chlorophyll content meter by clamping the test sample leaves. When the SPAD values were collected, the collection points avoided the leaf veins, and the four leaves were collected a total of 10 times, then the average value was taken as the final SPAD value of the leaves in the plot. The SPAD-502 plus determines the absorption of leaves in the red and near-infrared bands and calculates the LCC, so the method of characterizing the LCC by SPAD is accurate^44^.

#### Leaf spectral data acquisition

Spectral data collection was performed simultaneously with chlorophyll determination, and in this study, grape leaf hyperspectral data were measured using a hand-held leaf clamp of the ASD Filed Spec Pro 2500 back-mounted field spectrometer. The instrument collects spectral ranges from 350 to 2500 nm, with band accuracy and spectral resolution adjusted to 1 nm. Each determination of leaf spectral reflectance before a whiteboard correction, the determination of the leaf flat placed under the spectral detector with its own light source for direct measurement, in different positions of the leaf to be measured, uniformly collected 10 times the spectral reflectance, take the average value as the final spectral reflectance of the plot, a total of 91 plots.

### Method

#### Spectral preprocessing

The raw spectra contain signals such as baseline drift and noise, and there is also spectral drift due to the sample size as well as environmental factors. In order to improve the modeling accuracy, the raw hyperspectral reflectance data were preprocessed^45^. SNV and MSC can effectively eliminate spectral differences due to different scattering levels, thus improving the relationship between spectra and data.

SNV is mainly used to eliminate the effects of solid particle size, surface scattering, and optical range variations on the NIR diffuse reflectance spectrum. The principle is to transform the raw spectral data into standard normally distributed variables. The spectral data at each wavelength point is first mean-centered, and the variance deflation is performed after, thus eliminating the common drift and scaling effects in spectral data^46^. The formula is as follows:

1 $$\:\stackrel{-}{x}=\frac{\sum\:_{i=1}^{m}{x}_{i}}{m}$$

2 $$\:{x}_{SNV}=\frac{x-\stackrel{-}{x}}{\sqrt{\frac{\sum\:_{i=1}^{m}{({x}_{i}-\stackrel{-}{x})}^{2}}{(m-1)}}}$$

Where: $\:{x}_{i}$ is the hyperspectral data, $\:\stackrel{-}{x}\:$is the average of all the spectra, which is considered as the “ideal spectrum”, and $\:m$ is the number of wavelength points, $\:i=\text{1,2}\dots\:m$.

MSC corrects for baseline drift and offset phenomena in spectral data through ideal spectra. A one-dimensional linear regression of the spectra of each sample against the average spectrum was performed to obtain the baseline translation and offset, which was subtracted from the derived translation and divided by the offset thereby correcting for it^47^. The formula is as follows:

3 $$\:{x}_{i}=k\stackrel{-}{x}+{b}_{i}$$

4 $$\:{x}_{MSC}=\frac{{x}_{i}-{b}_{i}}{k}$$

where: $\:{b}_{i}$ is the translation and offset of each spectrum and $\:k$ is the offset coefficient of the spectrum.

#### Feature selection

The LCC sensitive band ranges were calculated using the Pearson feature selection algorithm. The Pearson correlation coefficient provides a quick understanding of the linear correlation between the features and the corresponding variables with directionality and outputs ranging from − 1 to + 1. It can be a measure of the strength of the relationship between the variables, the closer to 0 the lower the correlation, and is a special type of covariance that is standardized by removing the effect of the magnitude of the variables on both sides^48^. Once the correlation coefficient has been calculated, the strength of the correlation of the variables can be determined by the following range of values: 0–0.2 (very weak correlation), 0.2–0.4 (weak correlation), 0.4–0.6 (moderate correlation), 0.6–0.8 (strong correlation), and 0.8-1 (very strong correlation). In this study, correlation coefficients of 0.6 or higher were used as feature selection criteria. The formula is as follows:

5 $$\:r=\frac{{\sum\:}_{i=1}^{n}({X}_{i}-\stackrel{-}{X})({Y}_{i}-\stackrel{-}{Y})}{\sqrt{{\sum\:}_{i=1}^{n}({X}_{i}-\stackrel{-}{X}{)}^{2}}\sqrt{{\sum\:}_{i=1}^{n}({Y}_{i}-\stackrel{-}{Y}{)}^{2}}}$$

where $\:{X}_{i}$ denotes reflectance, $\:Y$ denotes LCC, and $\:n$ denotes sample point, $\:i$=1,2…n.

XGBoost introduces improvements such as distributed computing and second-order Taylor expansion of the loss function. The integrated learning of multiple CART trees is achieved by gradient tree boosting using CART decision trees as sub-models^49^. By counting the number of times a feature has been used as a split node in all trees as feature weights, the calculation is done by counting the total number of times each feature appears as a split node in the constructed decision tree model, which can be simply expressed as:

6 $$\:{FeatureImportance}_{weight}\left(f\right)=\sum\:_{t=1}^{T}I\left(f\:is\:split\:in\:treet\right)$$

where $\:T$ is the total number of trees and $\:I$ is an indicator function that is 1 if feature $\:f$ is used as a split node in tree t and 0 otherwise.

RFE is based on a wrapper type feature selection strategy. The study uses the random forest model as a base tool to assess the importance of features. It iterates continuously and trains the model based on the current set of remaining features at each iteration, gradually eliminating relatively unimportant features by iteratively constructing the model and evaluating the feature importance^50^.

PCA is a multivariate statistical analysis method. When there is a strong correlation between features is, these redundant features can be recognized and removed. The original data is projected into a new coordinate system by linear transformation, which makes the new features orthogonal to each other, the related features are merged to extract the most important part of information, thus reducing data redundancy^51^.

#### Model building

**Fig. 2:**
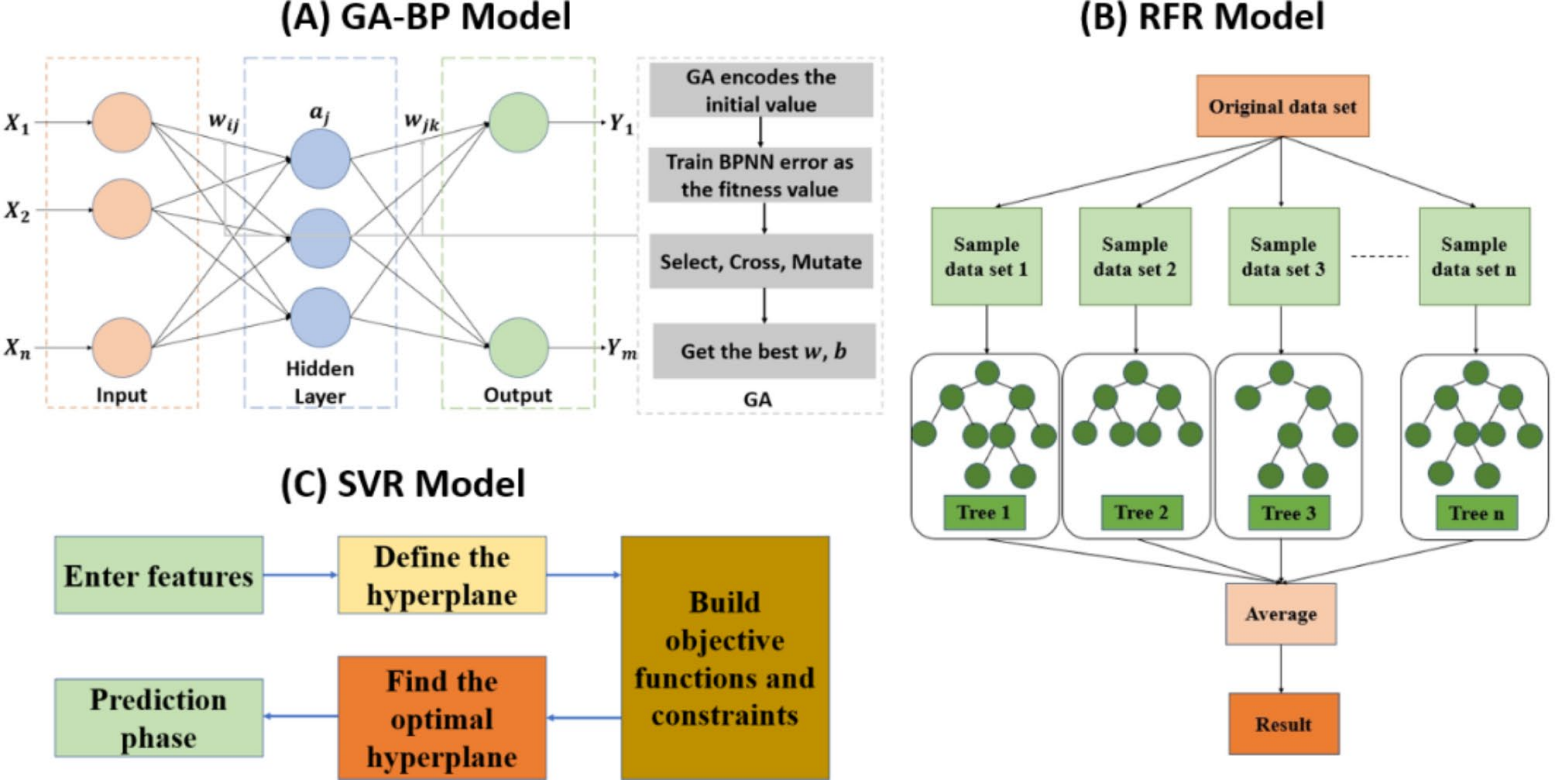
Model flow. (**a**) GA-BP model; (**b**) RFR model; (**c**) SVR model.

As shown in Fig. 2, BP takes the input signal features and maps them first to the Hidden Layer (realized by the activation function Sigmoid), and then to the output layer (linear transfer function) to obtain the desired output value. The error function is calculated by comparing the desired output value with the actual measured value, and then the error is back propagated. The weights w and threshold b of the BP network are adjusted by Gradient descent, and the process is repeated until the set error or the maximum number of iterations is met. Genetic algorithm is an optimization algorithm that simulates the process of biological evolution. It searches for optimal solutions in the solution space by simulating the operations of heredity, mutation and selection in nature. The algorithm has a global search capability to find the more optimal region in the complex solution space^52^. However, the initial weights and thresholds of the BP neural network are random, resulting in an unstable model effect, while the genetic algorithm can train the BP for optimization, correct the weights and thresholds in the network, reduce the network error, and make the model reach the optimum^53^.

RF is a classifier that contains multiple decision trees, which combines multiple base classifiers to achieve higher performance than individual classifiers and is widely used in classification and regression analysis. The basic idea of RFR is to collect a number of entries from a sample in order to train the model. Select the features based on the samples and construct a decision tree as a collection of base classifiers. Calculate the weight of each base classifier in the integration for more accurate results. According to the calculated weights to find the predicted mean value, through the run to generate a large number of decision trees to achieve a predetermined number of decision trees, so as to achieve the purpose of regression analysis. The algorithm has low computational complexity and can handle uncorrelated feature data, but the training and prediction time is long and not applicable to high-dimensional data^54^.

SVR models the regression process by finding an optimal hyperplane in two dimensions. Since this optimal hyperplane only considers points at the edges around the training set, it allows the model to effectively avoid overfitting of data points. Meanwhile, the complexity control parameter based on the reprojection error as a penalty term can well regulate the flexibility of the regression model. The model can effectively handle sublinear data with high accuracy and stability^55^. The basis function chosen in the study is radial basis function (RBF).

The three model parameters are designed as shown in Table 1:

**Table 1:** Model parameters.

Model	Parameter	Retrieve value
GA-BP	Hidden layer nodes	7
	Maximum number of iterations	1000
	Initial population size	40
	Error thresholds	$\:{10}^{-6}$
	Maximum number of evolutions	50
	Optimize the number of parameters	50
RFR	Number of decision trees	1000
	Minimum leaf number	3
SVR	Gamma	0.001
	Cost	1000

#### Model validation and evaluation

The coefficient of determination $\:{R}^{2}\:$standardized root mean square error NRMSE was chosen as a criterion for the predictive effectiveness of the regression model.

7 $$\:{R}^{2}=1-\frac{{\sum\:}_{i=1}^{n}{({y}_{i}-{x}_{i})}^{2}}{{\sum\:}_{i=1}^{n}{({y}_{i}-\overline{y})}^{2}}$$

8 $$\:RMSE=\sqrt{\frac{1}{n}\sum\:_{i=1}^{n}{({y}_{i}-{x}_{i})}^{2}}$$

9 $$\:NRMSE=\frac{RMSE}{\overline{y}}$$

Where: $\:{y}_{i}$, $\:{x}_{i}$, $\:\overline{y}$ is the mean of the predicted, measured, and measured chlorophyll content values, and n is the number of model samples.

## Results

### Hyperspectral data preprocessing analysis

**Fig. 3:**
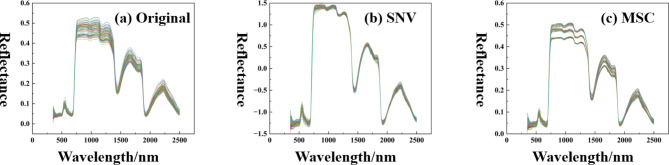
Hyperspectral and pre-processed images. (**a**) Original spectral curve; (**b**) SNV spectral curve; (**c**) MSC spectral curve.

As can be seen in Fig. 3, the trends of the reflectance spectral curves of grapevine leaves of different varieties are similar. This is due to the fact that green plant spectra are caused by the absorption of light by chlorophyll, other biochemicals, and cellular structures on the leaf surface, so their spectra are essentially the same. However, the local details of the curves vary considerably due to the different biochemical components among the different species. Spectral reflectance of grape leaves shows a large peak in the green and visible regions at 527–602 nm; In the green band from 500 nm, the absorption of the leaf decreases and the reflectance is increasing; Significant reflectance peaks at 551 nm, which is the non-absorbable part of the plant’s photosynthesis process, resulting in a strong spectral reflectance; The red band absorption valley is at 670 nm on the right side, after which the reflectivity rises steeply; The formation of a high reflective plateau in the near-infrared (NIR) band at 762–1096 nm may be due to the strong reflection of NIR light by the porous thin-walled cellular organization of the leaf blade; After 1285 nm, the spectral reflectance of the leaves starts to decrease; Near-infrared absorption valley at 1445 nm on the right side; The other two peaks were at 1588–1737 nm and 2118–2285 nm in the mid-infrared region, while the absorption valley in the mid-infrared band was at 1924 nm.

The trends of the spectral reflectance curves of grape leaves corresponding to different SPAD values were basically the same. The chlorophyll content of grape leaves shows a tendency to increase and then decrease with the period of fertility, due to the differences in the growth cycle between the two sites. During the berry growing season, leaves undergo sufficient photosynthesis to produce organic matter, and as large amounts of chlorophyll are synthesized, the chlorophyll content of the leaves also increases. At maturity, leaves begin to senesce, and chlorophyll begins to break down with transfer to be synthesized in new leaves, resulting in lower leaf chlorophyll content. Following the increase in chlorophyll levels, leaves with lower chlorophyll content had the highest reflectance in the visible range and the lowest reflectance in the near-infrared band. The most obvious change in leaf spectral reflectance in the visible region was near 550 nm, and the most obvious change in leaf spectral reflectance in the near-infrared band was near 750 nm, suggesting that there is a strong correlation between the spectral reflectance of grape leaves and chlorophyll content. Chlorophyll content inversion can be carried out through the law of change of spectral characteristics, to obtain the grape growth information, so that according to the real-time state of reasonable and timely fertilization to ensure that the grapes have a better growth trend.

Due to the large span of hyperspectral data, 3b and 3c are the SNV and MSC preprocessing methods, and it can be seen that the two methods eliminate the large gaps, removing the spectral differences due to the different scattering levels, and thus enhancing the correlation between the spectra and the data.

### Feature selection

**Fig. 4:**
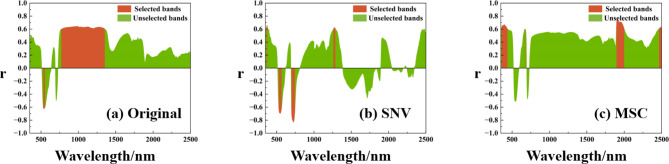
Pearson correlation analysis to determine the LCC sensitive spectral range. (a) Original hyperspectral sensitive band range; (**b**) SNV hyperspectral sensitive band range; (**c**) MSC hyperspectral sensitive band range.

Figure 4 represents the Pearson correlation analysis curves of the grape LCC with the hyperspectral data under different pre-processing, where the red sectors indicate the range of sensitive bands selected out. The sensitive bands of the raw hyperspectral data are mainly concentrated around 550 nm and 770–1350 nm, and the correlation is significantly negative for 550 nm and significantly positive for 770–1350 nm. This is due to the gradual formation and deepening of the red band absorption valley as the LCC content increases with leaf growth. The reflection peaks in the 800 nm near-infrared band are the result of multiple scattering of light inside the blade, where scattering of solar radiation by the blade dominates in this band range. Light entering the leaf is scattered multiple times between the cell walls, causing an increase in the probability that the light will again pass through the upper epidermis of the leaf and be picked up by the sensor, that is an increase in reflectivity. After the raw hyperspectral data are processed by SNV, the sensitive bands are mainly concentrated around 370 nm, 550 nm, 720 nm and 1270 nm. As can be seen from Fig. 4b, although the number of sensitive bands is reduced, the preprocessed data with LCC sensitivity is enhanced, especially near 720 nm where r reaches the − 0.8 highly significant level. The sensitive bands after MSC treatment are concentrated around 400 nm, 1950 nm and 2450 nm, and all these ranges show positive correlation with LCC.

**Fig. 5:**
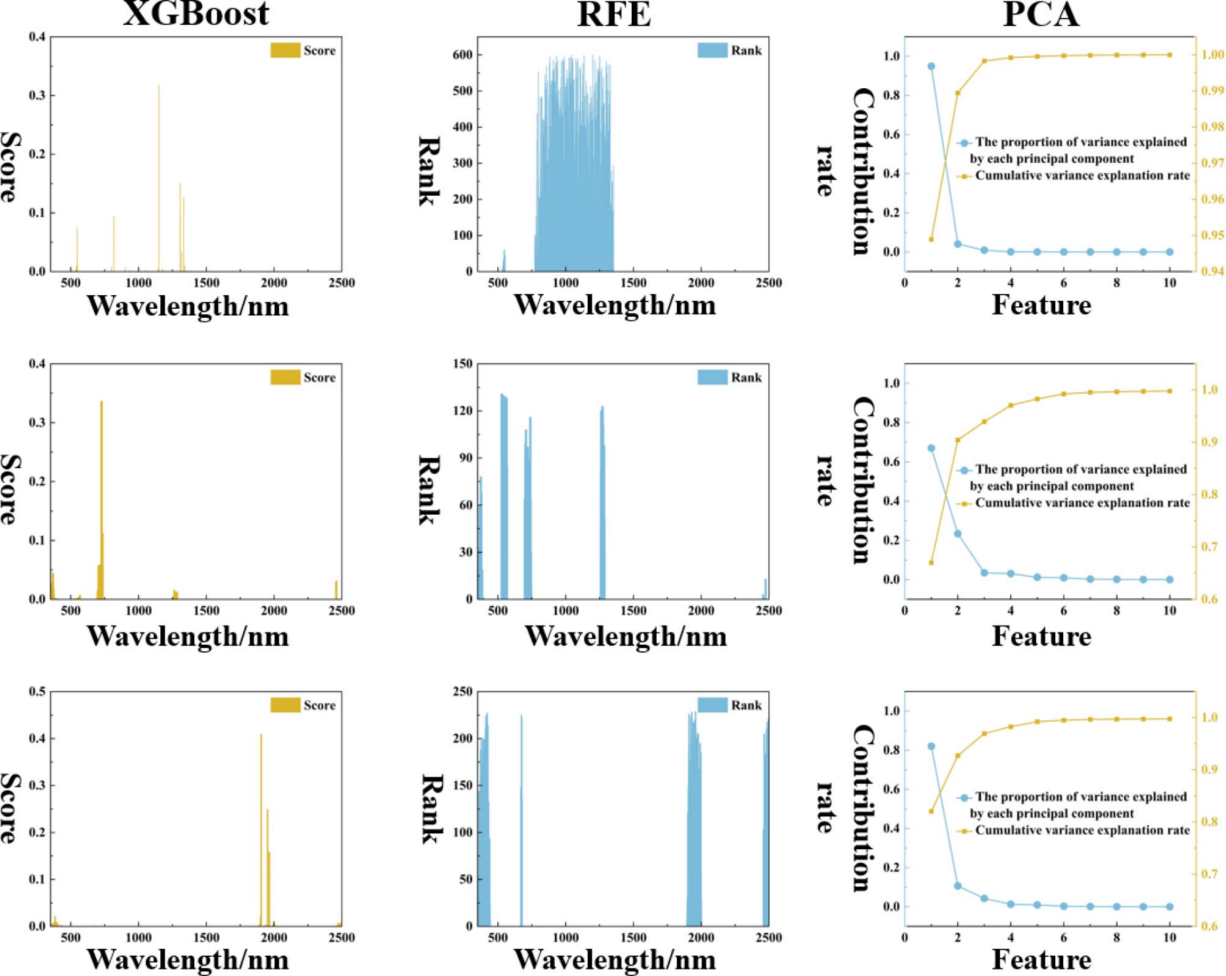
XGBoost, RFE, PCA feature extraction for sensitive bands with different preprocessing.

The sensitive bands screened by Pearson can be directly used as model inputs, but some sensitive bands are still highly correlated with each other, and it is crucial to improve the computational efficiency of the model and simplify the model structure while maximizing the retention of spectral information. As shown in Fig. 5, based on the sensitive spectral range, this study uses XGBoost, RFE and PCA to extract sensitive features as model inputs. The number of sensitive features set by XGBoost and RFE is 10, and the cumulative contribution of features set by PCA is 0.99. The score in XGboost indicates the feature importance, the larger the score, the stronger the feature importance. The screened sensitive bands are concentrated near 1100 nm, 700 nm and 1900 nm under different preprocessing methods, respectively. Rank in RFE is the feature importance ranking, the lower the Rank value, the stronger the feature importance. The screened features exist in each sensitive spectral range, and the method retains the spectral information to a greater extent, which is conducive to improving the model accuracy. PCA can calculate the contribution of mapping features, and the number of sensitive bands screened is 3, 6, and 5 under different preprocessing, respectively. The figure shows that the first feature contribution of the original spectra is much larger than that of the pre-processed spectra, indicating that there is a large amount of redundancy and error between the original spectra, highlighting the importance of pre-processing in hyperspectral remote sensing modeling.

### GA-BP, RFR and SVR model analysis

After the optimal features were obtained by feature screening through different preprocessing methods, the GA-BP, RFR and SVR models were used to predict and analyze the grape LCC. To improve the accuracy of the model, the data were normalized to eliminate differences in magnitude between different features. The dataset was divided in a 7:3 ratio and $\:{R}^{2}$ and NRMSE were used as model evaluation metrics. Table 2 represents the evaluation coefficients of LCC prediction models with different preprocessing and feature selection methods combined with regression models. the larger $\:{R}^{2}$ the more accurate the model is, the better the regression effect is, and the smaller the value of the NRMSE index indicates that the difference between the predicted value and the real value of the model is smaller, and the better the model is in predicting LCC.

**Fig. 6:**
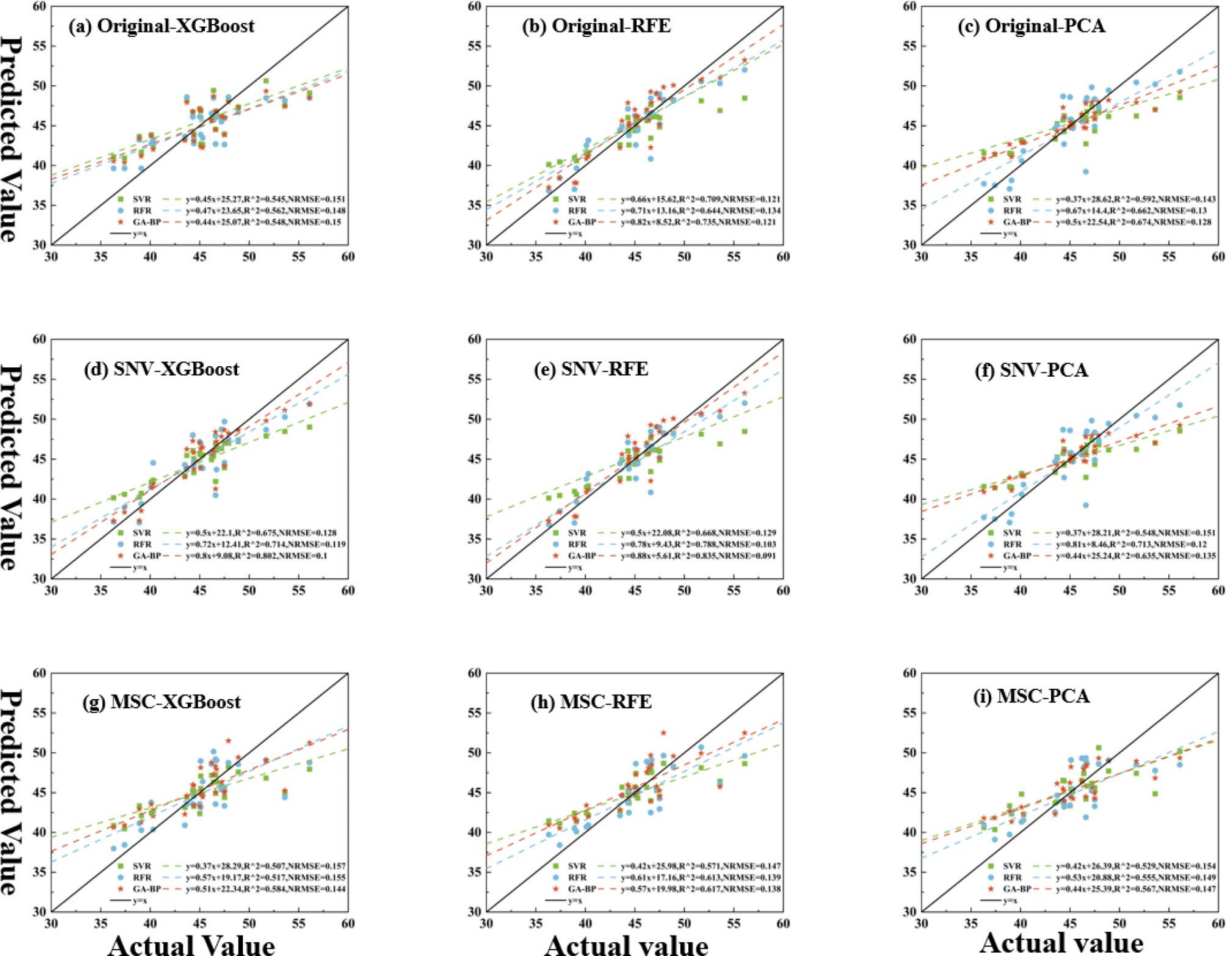
LCC fitting curves for different preprocessing, feature selection methods combined with regression models.

As shown in Fig. 6, the original spectra are all modeled better than XGBoost and worse than the RFE model after PCA dimensionality reduction. In contrast, the PCA modeling effects all performed poorly under different preprocessing effects. This may be due to the superior effect of PCA on the dimensionality reduction of the raw spectra, as the raw hyperspectral model is more in line with the physical mechanism, the features mapped are more representative and the reduction in dimensionality has a lower overall impact on the model. RFE shows good performance across preprocessing and models, while XGboost performs poorly. This is due to the differences exhibited by the two in feature screening methods, where RFE builds the model iteratively, eliminating the least important feature or features based on feature importance in the model at each iteration until a specified number of features or other stopping conditions are reached. This method is more suitable for situations where the number of features is high, especially when it is not clear which features are really helpful to the model, allowing the model to focus more on the really valuable features, thus reducing the risk of overfitting and allowing the model to make more accurate predictions on new data. Whereas XGBoost tends to select features that appear more frequently in the dataset when constructing a decision tree. This can lead to some low-frequency but actually valuable features being overlooked. And when there are complex correlations between the features, XGBoost may not be able to distinguish and select them well, so the bands selected by XGBoost are more concentrated in 3.2, and RFE covers all sensitive band ranges.

And in terms of modeling, GA-BP is almost all due to the rest of the models. Thanks to the GA algorithm, the model has a stronger global search capability, which makes the output of the neural network closer to the real value by continuously evolving the population, jumping out of the local optimum and finding more suitable parameters. The model generalization ability is also enhanced, and GA - BP enables the neural network to better balance the degree of fitting to the training data and the prediction ability to the unknown data by optimizing the parameter combinations during the training process. Thus, among all models, SNV-RFE-GA-BP achieved the optimal results, where$\:\:{R}^{2}$=0.835 and NRMSE = 0.091. This method cross-sectionally compares the SVR and RFR models of the same preprocessed SNV, and the $\:{R}^{2}$ is improved by 0.167 and 0.047, and the NRMSE is reduced by 0.038 and 0.012, respectively. Longitudinal comparison of the same model GA-BP, with different preprocessing Original and MSC, improved $\:{R}^{2}$ by 0.287 and 0.218, and reduced NRMSE by 0.03 and 0.047, respectively.

**Table 2:** Model performance.

	Model	SVR		RFR		GA-BP	
Pretreatment	Feature Selection	$\:{R}^{2}$	NRMSE	$\:{R}^{2}$	NRMSE	$\:{R}^{2}$	NRMSE
	XGBoost	0.545	0.151	0.562	0.148	0.548	0.15
Original	RFE	0.709	0.121	0.644	0.134	0.735	0.121
	PCA	0.592	0.143	0.662	0.13	0.674	0.128
	XGBoost	0.675	0.128	0.714	0.119	0.802	0.1
SNV	RFE	0.668	0.129	0.788	0.103	0.835	0.091
	PCA	0.548	0.151	0.713	0.12	0.635	0.135
	XGBoost	0.507	0.157	0.517	0.155	0.584	0.144
MSC	RFE	0.571	0.147	0.613	0.139	0.617	0.138
	PCA	0.529	0.154	0.555	0.149	0.567	0.147

**Fig. 7:**
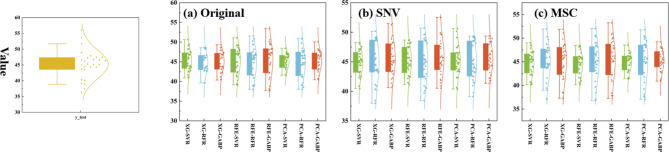
Distribution of LCC projections.

Figure 7 represents the different model LCC prediction boxplots and normal distribution curves used to determine if the models differed significantly due to assessment capabilities. It can be seen that the different models are ideal for predicting the LCC in the range of 40–50, but when the LCC is too high or too low, the enhancement of the model effect by the different preprocessing and feature selection methods is not significant. The SVR model was the least effective, with all predictions centered in the 40–50 range. This is due to the fact that SVR is the closest distance from the data to the optimal hyperplane by finding the optimal hyperplane, and the LCC is mainly concentrated in the range of 40–50, so the results are ideal in this range, and the performance of the model suffers when it goes beyond this range. After preprocessing, the GA-BP model is improved in out-of-range effect. The effect of noise and baseline drift is somewhat eliminated from the preprocessed data, and optimizing the weights of the neural network through GA enables the network to better fit the intrinsic patterns of the data. Using the global search and selection mechanism of GA to find a more representative combination of weights enables the neural network to better extract the essential features, thus improving the prediction accuracy on unseen data and enhancing the generalization ability of the network.

## Discussion

Leaf chlorophyll content is an important indicator of plant growth and development, and many researchers have conducted studies related to remote sensing estimation of leaf chlorophyll content^56,57^. Rapid prediction of grape LCC based on hyperspectral technology is conducive to large-scale and accurate monitoring of grape growth and improvement of fruit yield and quality.

Hyperspectral is high-dimensional data with strong inter-data correlation and a lot of redundancy and noise interference. Therefore, effective preprocessing and correlation analysis of spectral data to extract the characteristic bands can significantly reduce the model complexity and realize the simplification of hyperspectral data models^58^. Grape LCC is mainly reflected in the visible wavelength band, mainly due to the different absorption and reabsorption of photons by plant leaves with different chlorophyll contents. Based on the Pearson algorithm to extract the characteristic bands, the in situ hyperspectral of grape leaves reached significant correlation with LCC near 550 nm and 770–1350 nm, which is in line with the general characteristics of the green vegetation spectra, and is consistent with the conclusions of^59^ on the correlation analysis between leaf spectral reflectance and SPAD values.

After the raw hyperspectral data are processed by SNV, the sensitive bands are mainly concentrated around 370 nm, 550 nm, 720 nm and 1270 nm. Although the number of sensitive bands is reduced, the preprocessed data with LCC sensitivity is enhanced, especially near 720 nm where r reaches the − 0.8 highly significant level. The sensitive bands after MSC treatment are concentrated around 400 nm, 1950 nm and 2450 nm, and all these ranges show positive correlation with LCC. It is shown that preprocessing can effectively improve the correlation between the spectra and the data, consistent with the finding of^60^ those preprocessing methods can achieve effective spectral domain adaptation.

In this study, by comparing the three models, GA-BP, RFR and SVR, it was found that the BP neural network model based on GA optimization has better estimation ability in fitting the training samples and testing the test samples of chlorophyll values of grape leaves. After the original spectra are dimensionalized by PCA, the model is better than XGBoost, but worse than RFE model, and the PCA model is not so good under different preprocessing. The reason is that although PCA is effective in downscaling the original spectra, the original hyperspectral model conforms to the physical mechanism and its mapping features are more representative, and downscaling has little effect on the overall effect. This is consistent with the conclusion of^61^ that the first three principal components in the in situ hyperspectral were extracted by PCA and that the bands with the largest contribution from each should be unlikely to produce bands close to each other. RFE performs well in different preprocessing and different models, and XGBoost performs poorly, which stems from the different feature screening methods of the two. RFE reduces the risk of overfitting and makes predictions more accurate by iteratively removing unimportant features through iterative modeling, depending on the importance of the features, for situations where there are a large number of features and it is not clear which features are useful. Sun et al. used the RFE method to select the optimal wavelength and demonstrated that the method not only ranked the features according to their importance to maize yield, but also maintained the interpretability of the spectral data^62^. While XGBoost tends to select features with high frequency when constructing the decision tree, it is easy to ignore low-frequency but valuable features, and it is difficult to differentiate the selection in the face of complex correlation between features, so its selection of bands is more concentrated, and the RFE can cover the range of all sensitive bands.

In terms of modeling, GA–BP is mostly superior to other models. Thanks to the global search ability of the GA algorithm, it can go beyond the local optimum to find more suitable parameters, make the neural network output closer to the real value, enhance the generalization ability, and balance the ability to fit the training data with the ability to predict the unknown data. This is consistent with the conclusion of^63^ that GA and BP are used to create a prediction model and optimize the network parameters thus improving the learning efficiency and accuracy.

After statistical analysis of the model results, it was found that the accuracy was not satisfactory when the LCC was outside the range of 40–50, and even though the LCCs of different fertility periods were mostly concentrated in this range, the outliers were difficult to avoid due to the presence of manual errors during sampling. Therefore, in the future, when setting up experiments, we can consider eliminating the outliers so as to improve the accuracy of the model, or introducing a deep learning model to further mine the data features so as to improve the prediction performance.

## Conclusions

The hyperspectral-based grape LCC determination technique covers both wine grapes and table grapes categories in different periods, thus improving the model coverage and generalizability. In this study, grapevine leaf spectra and LCC were collected during three key reproductive periods, Cabernet Sauvignon (2022.08.19, ripening) and Sunny Rose (2023.08.23 and 2023.11.04, berry growth and ripening), to reveal the spectral response characteristics of the grapevine LCC using standardization by variables (SNV) and multiple far scattering correction (MSC) preprocessing variations. The sensitive spectral range was determined by the Pearson algorithm, and the sensitive bands of the raw hyperspectral data were mainly concentrated in the vicinity of 550 nm and 770–1350 nm; after SNV processing, the sensitive bands were mainly concentrated in the vicinity of 370 nm, 550 nm, 720 nm, and 1270 nm; and the sensitive bands of the MSC processing were concentrated in the vicinity of 400 nm, 1950 nm, and 2450 nm. Sensitive features were further extracted and redundant variables were eliminated using XGBoost, RFE, and PCA in this range. Three regression models based on GA-BP, RFR, and SVR were constructed to estimate grape LCC. A SNV-RFE-GA-BP framework for predicting hyperspectral LCC in grapes is proposed, where $\:{R}^{2}$=0.835 and NRMSE = 0.091. The study showed that the framework has a high potential for efficient detection of LCC in different categories of grapes, and the related research techniques can also be useful for the rapid monitoring of biochemical indicators related to grapes or other fruit crops.

## Data Availability

The datasets used and/or analysed during the current study available from the corresponding author on reasonable request.
